# An Intelligent Human–Unmanned Aerial Vehicle Interaction Approach in Real Time Based on Machine Learning Using Wearable Gloves

**DOI:** 10.3390/s21051766

**Published:** 2021-03-04

**Authors:** Taha Müezzinoğlu, Mehmet Karaköse

**Affiliations:** Department of Computer Engineering, Firat University, 23200 Elazig, Turkey; tmuezzinoglu@firat.edu.tr

**Keywords:** human–UAV interaction, wearable technologies, Internet of Things (IoT), human–computer interaction, smart systems

## Abstract

The interactions between humans and unmanned aerial vehicles (UAVs), whose applications are increasing in the civilian field rather than for military purposes, are a popular future research area. Human–UAV interactions are a challenging problem because UAVs move in a three-dimensional space. In this paper, we present an intelligent human–UAV interaction approach in real time based on machine learning using wearable gloves. The proposed approach offers scientific contributions such as a multi-mode command structure, machine-learning-based recognition, task scheduling algorithms, real-time usage, robust and effective use, and high accuracy rates. For this purpose, two wearable smart gloves working in real time were designed. The signal data obtained from the gloves were processed with machine-learning-based methods and classified multi-mode commands were included in the human–UAV interaction process via the interface according to the task scheduling algorithm to facilitate sequential and fast operation. The performance of the proposed approach was verified on a data set created using 25 different hand gestures from 20 different people. In a test using the proposed approach on 49,000 datapoints, process time performance of a few milliseconds was achieved with approximately 98 percent accuracy.

## 1. Introduction

The Internet of Things (IoT) refers to large communication networks that effectively manage and use data from objects or physical activities employed in daily life. Spatially distributed sensors and nodes, each with a transceiver, can be monitored, detected, or triggered to communicate within a network using a controller [1,2,3]. Wearable technologies are an important complement to the concept of the internet of things. Today, wearable technologies can perform desired tasks or events with the help of devices found in many different configurations on many parts of the body [4,5]. These devices include gloves, bracelets, bands and helmets, contact glasses, headphones, globes, digital pens, smart clothing, jewelry, and even tattoos [6,7].

In this study, we discuss the issue of human–UAV interactions using wearable technologies. The ability of UAVs to reach places that humans or other robots have difficulty accessing and perform their tasks makes UAVs unique [8]. Unmanned airborne systems (UASs), especially small unmanned airborne systems (S-UASs), are used in military reconnaissance, search, and intelligence work in areas that are not safe for humans, as well as in remote sensing areas; data collection is used due to its data storage, analysis, and decision capabilities [9]. Work performed in areas that are not safe for humans and difficult to access can be performed thanks to human–UAV interactions [10,11,12,13,14,15,16,17,18,19,20,21,22]. One of the areas where human UAV–interactions are used is computer-vision-based hand gesture recognition applications, which enable users to determine hand gestures using pattern recognition algorithms [23,24,25,26,27,28,29]. Although this method can detect a large number of movements, its efficacy can be affected by the variability of ambient lighting and possible obstacles in the line of sight since this method relies on computer vision. In addition, this method requires the installation of peripheral equipment (camera, lighting, etc.) beforehand to obtain better images. Since vision-based hand gesture recognition applications use computer vision techniques, using pattern recognition algorithms for users’ hand gestures entails a high cost, difficult setup, and excess equipment.

### 1.1. Background

The work we carried out includes the design of two wearable smart gloves for human–UAV interactions and classification methods for investigation results to define hand gestures that will enable UAV flight control. The hardware and software components of the study and the proposed system architecture are discussed in detail in the relevant subsections. Hu et al. [30] defined 10 dynamic movements for UAV motion controls. To recognize the designed movements through the hand gesture recognition system, the skeleton data collected from a Leap motion control device are transformed into two different data models. To train and test the deep learning neural network, samples were created from the data generated using approximately 9000 data from the system. The static test results of this study obtained an average accuracy of 96.7% in the scaled data sets of the 2-layer fully connected neural network, an average of 98.0% in scaled data sets of the 5-layer fully connected neural network, and an average of 89.6% in the scaled data sets. Labazanova et al. [31] acquired right-hand samples from six people and enabled the user to intuitively and safely control a drone herd in virtual reality environments with a wearable tactile glove. Signal samples were analyzed using ANOVA, and the experimental results were 86.1% successful. Lee et al. [32] obtained data with the aid of Gsr and IMU sensors from eight people—including two women and six men—with 4–10 years of driving experience using the glove designed in the study. Using the obtained data, the authors defined a stress classifier model using an SVM to distinguish between stressful and stress-free driving situations. Song et al. [33] proposed a smart glove capable of appropriately sensing finger pressures and bending angles to measure the kinetic and kinematic parameters of the player during right-hitting and backhand handling in badminton. When designing the smart glove, the authors used an Arduino board, 21 pressure sensors, and 11 flexibility sensors and then visualized the parameters measured by these sensors to analyze the different grip movements. Using the measured joint angles and pressure data, 10,000 datapoints were obtained, and a method for visualizing these data on a computer was proposed. Benatti et al. [34,35] developed an sEMG-based hand gesture recognition system using IMU sensors. In a previous study, Benatti used the SVM classification method in seven motion recognition systems with the data received from four people and achieved approximately 90% success. In another study, Benatti tested a wearable electromyographic (EMG) motion recognition application based on a hyper-dimensional computational paradigm running on a parallel ultra-low-power (PULP) platform, with the ability to perform online learning with the system; this study was done with 10 people and detected 11 gestures with an average accuracy of 85%. Chowdhury et al. [36] presented a rehabilitation protocol involving the PP phase followed by the MP phase by designing a hand exoskeleton and brain–computer interface, as combining mental practice (MP) and physical practice (PP) in post-stroke rehabilitation largely provided positive rehabilitation outcomes. In this study, the authors conducted a 6-week clinical study on four stroke patients. In the first stage, the usability of the system based on changes in functional motor recovery, grip strength (GS), and Arm Test Action Research scores were determined by considering mood, fatigue, and motivation parameters on a visual analog scale (VAS). Zhu et al. [37] designed a tactile force-fed smart glove with triboelectric-based finger flexure sensors, palm shift sensors, and piezoelectric mechanical stimulators. The detection of multi-directional bending and shifting states was used for tactile mechanical stimulation with piezoelectric chips to realize human–machine interactions via self-generated triboelectric signals under various degrees of freedom on the human hand. In this study, the authors achieved object recognition with 96% accuracy by using the SVM machine learning technique with smart education. Berezhnoy et al. [38] proposed an approach to create a hand gesture recognition-based control interface and provide UAV control with a wearable glove system consisting of an Arduino nano microcontroller, an IMU, and flex sensors. In this study, the authors took right-hand samples from six people and performed motion capture and evaluation with fuzzy C-means (FCM) clustering and realized motion data acquisition via fuzzy membership functions to classify the data. Jiang et al. [39] proposed a real-time motion recognition wristband able to recognize four surface motions with eight air movements and levels using sEMG and IMU sensors. Using the test data from 10 healthy individuals, in the motion recognition test, an air movement accuracy rate of 92.6% and surface movement accuracy rate of 88.8% were observed. Chen et al. [40] implemented a promising IoT healthcare application for home hand rehabilitation. An ARM microcontroller, force sensors, and flex sensors were used to create a pair of gloves: a sensory glove and a motor glove. Three different machine learning techniques were also tested to classify the movement types. Accuracy rates of 99.07%, 97.04%, and were obtained for the SVM, KNN, and DT methods, respectively. The proposed system defines 16 types of finger movements with an average accuracy of about 93.32%. Yang et al. [41] proposed a system that can be used as an educational tool to facilitate the rehabilitation process for post stroke patients. A smart wearable armband was designed to recognize hand gestures. This armband receives signals from three different users via an Arduino and sEMG sensor. Classification complexity estimation algorithms are classified via CCEA and principal component analysis (PCA) to analyze and distinguish the characteristics of different hand gestures. According to the verification results, all nine movements were successfully identified, with an average accuracy of up to 96.20%. The many applications in the literature show differences in terms of their usage purposes and methods.

**Figure 1:**
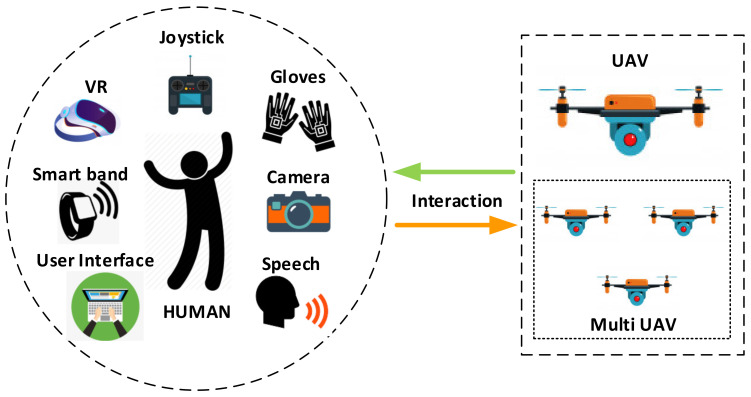
Methods used for UAV control in single or multiple human–UAV interactions.

Wearable technologies are mostly used in medical applications, fitness and health, knowledge, and UAV applications. Based on the literature review, the sensor types and methods used in their application are classified and shown in Table 1. With the acceleration of research and development studies on UAVs, this technology has begun to be used in many new fields other than military and defense [42]. These developments have enabled UAVs to move towards human–UAV interactions, rather than environmental surveillance functions. In regions affected by natural or man-made disasters, damaged areas need to be efficiently restored [21]. In a large-scale damaged transmission network, it is important to use unmanned aerial vehicles (UAVs) to investigate preliminary unexpected/unidentified failures [13]. As UAVs can work alone or move in a herd, they have achieved successful results in challenging missions [11,29]. Work that needs to be performed in unsafe and difficult-to-access areas can now be done using human–UAV interactions [11,12,13,14,15,16,17,18,19,20,21,22,43]. Human–UAV interactions are only used for information exchange in some applications, while some applications employ physical contact or force exchange. As can be seen in Figure 1, this interaction can be performed using a software interface alone or by using physical [11], tactile [12], auditory [44], and human kinetics techniques or creating a specific rule table [20,21,22,23].

One of the areas where human–UAV interactions are used is computer-vision-based motion recognition applications that enable users to make predictions using pattern recognition algorithms such as hand gestures and body movements [23,24,25,26,27]. Motion recognition applications are generally costly—e.g., UAV cameras, CCD and micro cameras, Leap motion controllers, and Kinect. Moreover, the processing and return times are long because the data sizes are large. Although this method can detect a large number of movements, the method can be affected by the variability of ambient lighting and possible obstacles in the line of sight since this method relies on computer vision. Additionally, this method requires the installation of peripheral equipment (camera, lighting, etc.) beforehand to obtain better images. The installation of such systems requires a qualified human workforce as well as high-cost equipment. Therefore, the setup time for human–UAV interaction systems and the return times of these systems are increasing. UAV controls using wearable technology such as low-cost, simple gloves consisting of sensors and microprocessors are good examples of human–UAV interactions that are affordable and useful [28,38].

### 1.2. Contributions

This study features the design of two wearable smart gloves for human–UAV interactions and UAV steering actions used with multi-mode commands for classification methods to define hand gestures for providing UAV flight control. In our study, a method is proposed to define 25 different basic gestures and design a wearable pair of smart gloves that will provide flight control for a UAV and direct the UAV with correct and complete movements. A data set consisting of 49000 data in total was obtained from 20 different users consisting of men and women aged 22–63. The SVM classification test on the hand gesture data set had the highest accuracy rate with 98.02% success, while the best test time was 1.22 ms with the KNN classification algorithm.

This study makes to following scientific contributions:

- A multi-mode command structure was created, and successive commands were recognized.
- Hand gesture classification and recognition based on machine learning was realized.
- A mission planning algorithm was developed to manage the UAV simulation correctly.
- Accuracy rates as high as about 98 percent were achieved for real-time and effective use.

### 1.3. Organization

The reminder of the paper is organized as follows. Section 2 outlines the proposed method; in this section, the proposed method and algorithms for intelligent human–drone interactions are described based on machine learning using a pair of wearable gloves in real-time, and the parameters of the preferred classification algorithms are given. In Section 3, the overall system design, materials used in the study, the properties of these materials, and the hardware and software infrastructure are explained along with the experimental results. Here, the confusion matrix and success results are given for the scenarios created. Moreover, a comparison of the results with other studies in the literature is provided, and the advantages of the method are explained. In Section 4, the discussion section, studies in the literature are compared in terms of their methods for time sensing systems, and their advantages and disadvantages are discussed. In Section 5, the conclusions, this study’s success and advantages over previous work in the literature are outlined.

## 2. Proposed Method

**Figure 2:**
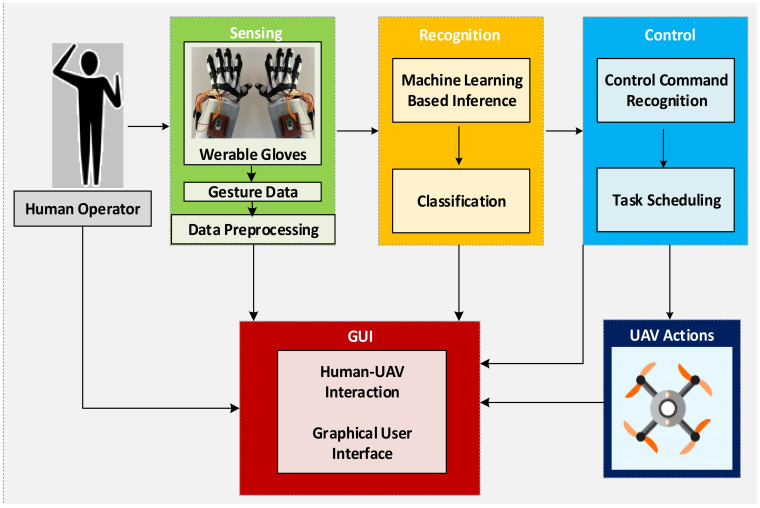
The proposed architecture for hand movement prediction and UAV guidance through an intelligent pair of gloves consisting of the sensor systems, the recognition system, the control system, the GUI (Graphical User Interface), and the part where the user controls the UAV’s movements.

In this study, we designed a flexible soft pair of smart gloves that can be worn comfortably by users of all ages and genders. With these smart gloves, signals received from the flex sensors and IMU sensors integrated on the gloves are received through the STM32 microprocessor and transferred to the host computer to detect hand movements correctly. The process of displaying, sensing, and converting these received signals into digital values is realized through the user interface we designed. To make this data meaningful and able to recognize hand movements, these data are transformed into a smooth data set that can be classified using four different ML algorithms. As a result of this classification, a real-time method is proposed to provide the UAV task control according to the commands determined by task scheduling. The proposed architecture for hand movement estimation and UAV command control operations using a smart pair of gloves is shown in Figure 2.

Flexibility sensors and the STM32 card connections of the IMU sensor were designed according to the pins, resistance values, and technical data specified in the datasheets to collect motion data from the human operator. In the study, the signals received from the flexibility sensors and IMU sensors were then transferred to the host computer via STM32. Through the GUI that we developed on the main computer, serial port connections between the glove computer were provided, and the signals were transferred to the host computer.

Gyro and acceleration sensor signals on the IMU, in addition to the flexibility sensors corresponding to hand gestures, are graphically shown in our application. These graphics can be followed in real-time during the application. To avoid distortion during the beginning and end of the motion, data from the first and last 3% of the signals received from the human operator were removed. The signals received in the gloves were pre-processed and converted into digital data. To create a data set of digital values and then teach the system in the classification section, saving was performed according to the location and file type determined through the application. A normalization process was carried out to purify these data of repetition, and then the data set to be used was obtained. For the data set obtained, 4 different classification algorithms were applied to make machine-learning-based inferences. The mission planning of the multi-mode commands corresponding to the hand gesture control definition class determined as a result of the classification process was transferred to the UAV simulation developed in the Unity environment via a GUI.

### 2.1. Normalization and Datasets

Before classification, data were collected by preserving the received data for 10 s after each movement to give accurate results from the sensors on the gloves. To ensure the quality and accuracy of the motion signals, 3% of the data belonging to the first and last parts of the received signals were removed, as the user had to clearly complete the hand movement. The purpose of this normalization process was to increase the success rate of the classification process and prevent false results by eliminating the end signal problems that may occur due to the electrical hardware structure in the designed system.

The normalization process (V′) is found by dividing the smallest (min(set)) and largest (max(set)) data in the data set (V) by the extracted result. The normalization process was applied separately for each hand movement in the data set. The formula for the function of the normalization process is provided as Equation (1):

V′ = V − min(set)/max(set) − min(set)(1)

**Figure 3:**
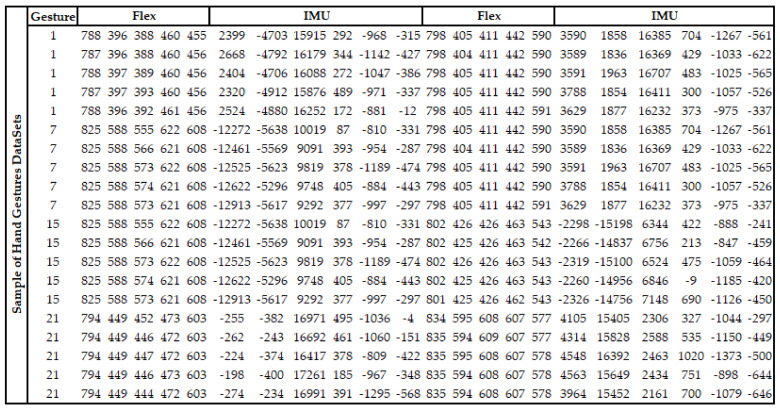
Sample data from the constructed hand gestures for four gestures.

In this study, a data set was created from the data obtained via flex and IMU sensors on a wearable smart glove. The data set used to recognize hand gestures consisted of 49000 data obtained from 20 different users between the ages of 22 and 63 (both men and women). Four sample gesture data (gesture 1, gesture 7, gesture 15, and gesture 21) shown in Figure 3.

### 2.2. Classification

These recorded data were transferred to the MATLAB Classification Learner environment and subjected to classification with the help of machine learning algorithms for training–testing processes. Four different ML algorithms were used for classification: decision tree (DT), naïve Bayes (NB), support vector machines (SVM), and K-nearest neighbors (KNN).

#### 2.2.1. Decision Tree

Decision Trees usually apply a set of rules that depend on one variable at a time. These trees divide the entrances into sections, arranging the detail at each level until reaching the end of the tree while also determining the leaf node that provides the final predicted tag. Since simple decisions are made at each stage, and it is not necessary to go through an entire tree to find the right class, decision tree classification is interpreted and executed extremely quickly [47].

Step 1. The data set is placed at the root of the tree.

Step 2. Divide the data set into subgroups. Subgroups are made so that each group contains data of the same value.

Step 3. Repeat steps 1 and 2 for each subset until you find leaf nodes on all branches of the tree.

- m: The number of states whose entropy is to be calculated
- $p_{i}$: *i* probability of condition
- $D_{1}$: dataset.

(2) $$Entropy\left(D_{1}\right)=-\overset{m}{\underset{i=1}{\sum }}p_{i\;}\;\log_{2}p_{i}$$

Information Gain (IG) = Entropy (S) − Entropy (Target)(3)

Step 4. The entropy of the target class is calculated as in Equation (2).

Step 5. Entropy must be calculated for each grade. Information Gain (IG) The entropy obtained by using the formula in Equation (3) is subtracted from the target entropy.

#### 2.2.2. Naïve Bayes

Naive Bayes is a machine learning algorithm and a classification technique. Since Naive Bayes is fast and based on Bayesian statistics, it is efficient at real-time forecasting. Most popular real-time models are based on Bayes statistics [48]. Naive Bayes works well when the resulting variable extends to more than one class. Since Naive Bayes works best with discrete variables, it tends to work well in such applications.

The Bayes formula is shown in Equation (4). Naïve Bayes aims to choose class c with the maximum probability. Argmax is the process used to find the class that gives the maximum value from a target function. In this case, the maximum class c is found using Equation (5):

(4) $$P(c\;|\;x)\;=\;\frac{P(x\;|\;c)}{P\left(c\right)P\left(x\right)}$$

(5) $$\;\;\;\;\;\;\;\;\;\;\;\;\;\;\;\;\;c=\;{\mathrm{argmax}}_{c}\left[P\left(c\right)*\;\overset{n}{\underset{i}{\prod }}P({x\;}_{i}\left|\;c\right)\right]$$

where P (c|x), the predictor (attribute) is the last probability of the given class (target), P (c), is the priority probability of the class; P (x|c), is the probability of the estimator given in the class; P (x), is the estimator’s previous probability and n, is the number of nodes.

#### 2.2.3. Support Vector Machine

Support Vector Machine is one of the most versatile controlled machine learning algorithms and is generally preferred when classifying medium- and small-sized data sets. With SVM, a great deal of fine-tuning is not required to achieve good results. SVM draws a boundary between the two classes in one plane to classify data for two classes. The place where this boundary will be drawn should be the furthest area from the elements of both groups. This determines how the border will be drawn. SVM aims to obtain the most suitable plane to separate classes from each other [49].

#### 2.2.4. K Nearest Neighbor

In the KNN (K-nearest neighbor) classification algorithm, a value of k is determined first. Provided this value is a positive integer, a smaller value is generally preferred. The K value should be chosen to look at the nearest k neighbors to look at when new data comes to the data set. The new value to be determined should be determined according to the majority value of k neighbors [50]. Often the Euclidean function is used to calculate the distance to the neighbor. For the calculation of the distances, the Euclidean distance formula is preferred for i and j points.

The Euclidean distance calculation formula is shown in Equation (6):

(6) $$\mathrm{Euclidean}\;\mathrm{Distance}\;i,j\;=\;\sqrt{\overset{n}{\underset{k=1}{\sum }}{\left(x_{ik}-y_{jk}\right)}^{2}}$$

where k is the number of neighbors closest to a point and n: is the neighbor number to look at.

In the classification process, each incoming datum for the distance calculation process is defined as a new point on the coordinate plane. The Euclidean distance is the square root of the sum of squared differences between the new instance (𝑥𝑖) and the existing instance (𝑦𝑗). This method is based on choosing the distance of each of the observations in the dataset from an observational value determined later and k number of observations with the smallest distance. After the distance values are calculated, the data are sorted, and the class that the incoming value belongs to is determined.

The parameters of the classification algorithms used in the proposed method are shown in Table 2. The success of these classification algorithms depends on the correct selection of these parameters. Parameters are very important, as they will affect uptime and accuracy rates.

As a result of the classification process, a hand gesture class of 25 gestures was determined. The mission planning for the movement corresponding to the multi-mode commands that will control the UAV specified in the proposed method for the specified class is shown in Table 3. The motion details of the UAV in the simulation environment are explained in the mission planning table.

## 3. Experimental Results

### 3.1. Experimental Setup

In this study, a wearable pair of smart gloves is designed, and a method to perform all the movements and tasks of the UAV to be managed without the need for a controller is proposed. Two gloves were designed to ensure the interactions between the UAV and the user, and a GUI was developed to correctly obtain the signals from the glove. Using this GUI, the signals received from the sensors used in the glove design can be observed and recorded, and the control inputs can be made correctly. Additionally, with this GUI, a data set was created by converting analog signals from the gloves into digital values. The data set obtained is very important, as it will directly affect the success in terms of accuracy rates during the training and testing of the system. The hardware and software components used in this study are explained in detail in the relevant subsections.

#### 3.1.1. Hardware Design

**Figure 4:**
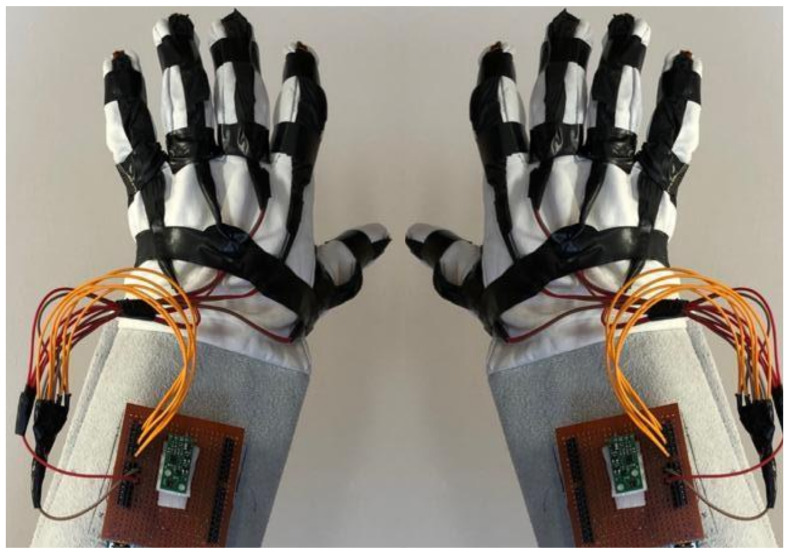
A pair of smart glove designs built with Flex and IMU sensors and an STM32 card.

For wearable smart glove design, a flexible glove that is comfortable to use was determined to be preferable. Our main goal in choosing gloves was to provide ease of use for volunteers of all ages and genders. Five flex sensors, one IMU, and one STM 32 microcontroller are installed in each glove to collect data safely and with good sensitivity. Flex sensors were sewn and fixed on the glove, corresponding to each finger. Each glove was covered with a flexible latex layer to protect against impacts and corrosion from the external environment. Our wearable smart glove is shown in Figure 4.

Flex sensors used in wearable smart glove design are important for detecting the bending movements of the fingers. Basically, the resistance values change depending on bending and twisting, which works with the logic of resistance. This sensor, also known as a bending sensor, has a resistance value of ~10 KΩ when in a flat state. During bending, the resistance will increase up to ~20 KΩ. These values may vary depending on the resistance used.

The size and technical features of the 4.4-inch-long flex sensors (Adafruit brand) used in the system are shown in Table 4.

The wearable smart glove design used an IMU (Multiple Sensor Unit) Card including an accelerometer, inertial meter, and gyroscope. The LSM6DS33 is a card combining a 3-axis gyro and accelerometer, a LIS3MDL 3-axis compass sensor, and a LPS25H digital barometer sensor. The IMU sensor, which can be operated using an input voltage between 2.5 and 5.5 V with its voltage regulator, provides 16-bit resolution. The basic features of the AltIMU-10 v5 sensor are provided in Table 4.

The STM32 microcontroller (STMicroelectronics International N.V., Geneva, Switzerland) has a 32-Bit ARM Cortex-M3 core. This card offers a powerful and economical solution in terms of usage and cost and supports simulation, downloading, and debug operations thanks to its SWD interface. An Epson brand 8M crystal is used to adjust the frequency system to 72 MHz, and the USART1 protocol is used to download programs or communicate. The technical features of the STM32 card used in the developed system are shown in Table 4.

The main computer used in our experimental environment works at a maximum frequency of 4.7 GHz and features an Intel-Core i7-9700 processor operating at the stock 3.0 GHz speed. This computer has 48 GB of RAM and supports 64-bit architecture, with Windows 10 used as the operating system.

#### 3.1.2. The Software Development

The motion recognition software used in our study involves three main steps: signal preprocessing, the learning–test process for classification, and motion recognition. With the help of the designed smart glove, analog data from the flex and IMU sensors are received via the STM32 card. The received signals are then transformed into digital value parameters by transferring the signals to the computer environment via a USB connection. These values are transformed into a data set using a GUI developed in Microsoft Visual Studio C#. The signals can be followed one by one from the signal control center in the GUI for each movement.

A normalization process was applied to the data recorded using the GUI. The Classification Learner Toolbox was used to classify this data set in the MATLAB 2020a program. The hand gesture recognition process was performed using four different machine learning classification algorithms;

- Decision tree (DT),
- Naïve Bayes (NB),
- Support vector machines (SVM),
- K-nearest neighbors (KNN).

Cross-validation is used in education and measured by the recommended value. In the proposed method, the performance of the classifiers is obtained by crossing the data not used in education 10 times. After running 100 iterations, the accuracy, precision, recall, geometric mean, and F-Score results can be obtained for each classification. According to these results, we can estimate to which class the movement belongs. If, as a result of the classification, the class to which the movement belongs is not one of the determined movements, data retrieval is performed again. If the motion estimation belongs to one of the classes determined as a result of the classification, motion control is again performed using the UAV simulation in the Unity environment.

The accuracy (CACC), unweighted mean precision (UAP), unweighted mean recall (UAR), unweighted average precision (UAP), F measure (F1), geometric mean (GM), and root mean square error (RMSE) parameters were calculated. The mathematical expressions required to calculate these parameters are given in Equations (7)–(12), respectively.

(7) $$CACC=\frac{TP_{c}+TN_{c}}{TP_{c}+TN_{c}+FP_{c}+FN_{c}},c=\left\{1,2,\ldots NC\right\}$$

(8) $$UAP=\frac{1}{NC}\overset{NC}{\underset{c=1}{\sum }}\frac{TP_{C}}{TP_{c}+FP_{c}}$$

(9) $$UAR=\frac{1}{NC}\overset{NC}{\underset{c=1}{\sum }}\frac{TP_{C}}{TP_{c}+FN_{c}}$$

(10) $$F1=\frac{2*\left(UAP*UAR\right)}{UAP+UAR}$$

(11) $$GM=\sqrt[{NC}]{\overset{NC}{\underset{c=1}{\prod }}\frac{TP_{c}}{TP_{c}+FN_{c}}}$$

(12) $$RMSE=\sqrt{\frac{1}{N}\overset{N}{\underset{i=1}{\sum }}{\left(D_{predicted}-D_{true}\right)}^{2}}$$

where $TP_{c}$ = number of correct positives, $TN_{c}$ = number of true negatives, $FP_{c}$ = number of false positives, $FN_{c}$ = number of false negatives, NC = number of classes, $D_{predicted}$ = the predicted value, $D_{true}$ = the true value and N = total number.

### 3.2. Results

To detect hand gestures, the UAV routing process was carried out by displaying the signals from the sensors in response to the commands given over the two smart gloves we developed and estimating the motion through the classification methods of the data. There should be a control center or a control mechanism used to control the flight movements of the UAV to direct it easily. This control mechanism can be a remote, a joystick, or a telephone.

In this study, a pair of smart gloves were designed with wearable technology for use in the experimental environment. With smart glove, gesture types consisting of movements and commands can be determined to direct the UAV correctly without needing a control mechanism. The gesture types are designed in such a way that each hand acts like a control mechanism. Table 5 shows the hand movement classes used in the classification process and the movement types describing the direction and axis in which each movement should move the UAV.

**Figure 5:**
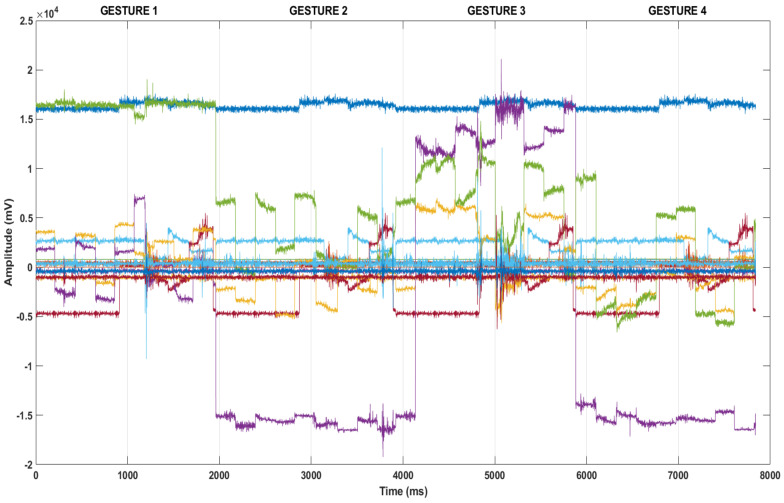
An example of a signal graph that occurs when 4 consecutive hand movement scenarios occur for approximately 2000 ms.

After connection is made with smart gloves from the serial port by pressing the connect button, the application starts to run when the start button is clicked in the GUI. Data from the Flex and IMU sensors are displayed in the instant data display area. The signals received from the sensors on the two gloves for each movement involve 22 parameters. Approximately 2000 ms sampling signal graphics for four consecutive sample gestures in the selected scenario are shown in Figure 5 on the amplitude time axis.

The sampling frequency of the data from the Flex sensors and the IMU sensor is planned to be 100 kHz. The signals received for sampling were sufficient to provide a clear understanding of the hand movement. This data set consists of 49,000 sensor data points taken from the 20 different users consisting of men and women aged 22–63. After the desired amount of data are obtained, if desired, the data alone can be monitored, or the save location can be specified and saved in the desired format via the save button.

**Figure 6:**
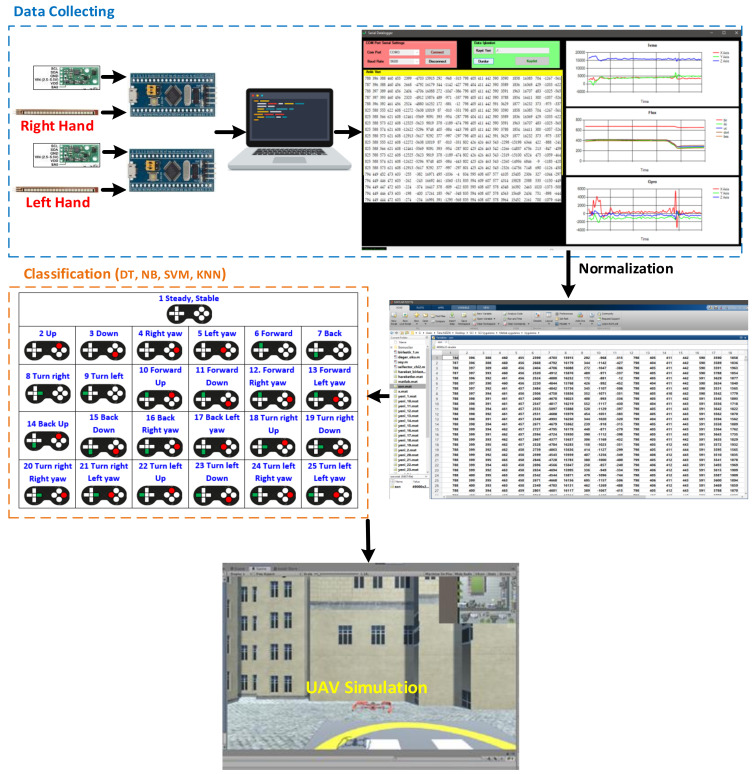
The stages of classifying hand gestures via a pair of smart gloves that can be used in real time with the help of machine learning algorithms and controlling the UAV in a simulation environment.

A normalization process was then carried out to remove erroneous or repetitive data from the data set. After the normalization process, the data set was subjected to 4 different classification methods. As a result of this classification, we determined to which class the 25 different hand gestures belonged. The UAV flight guidance command corresponding to this class was directly transferred to the UAV simulation in the Unity environment. The operating steps for the UAV guidance system based on application screen hand gesture recognition are shown in Figure 6.

**Figure 7:**
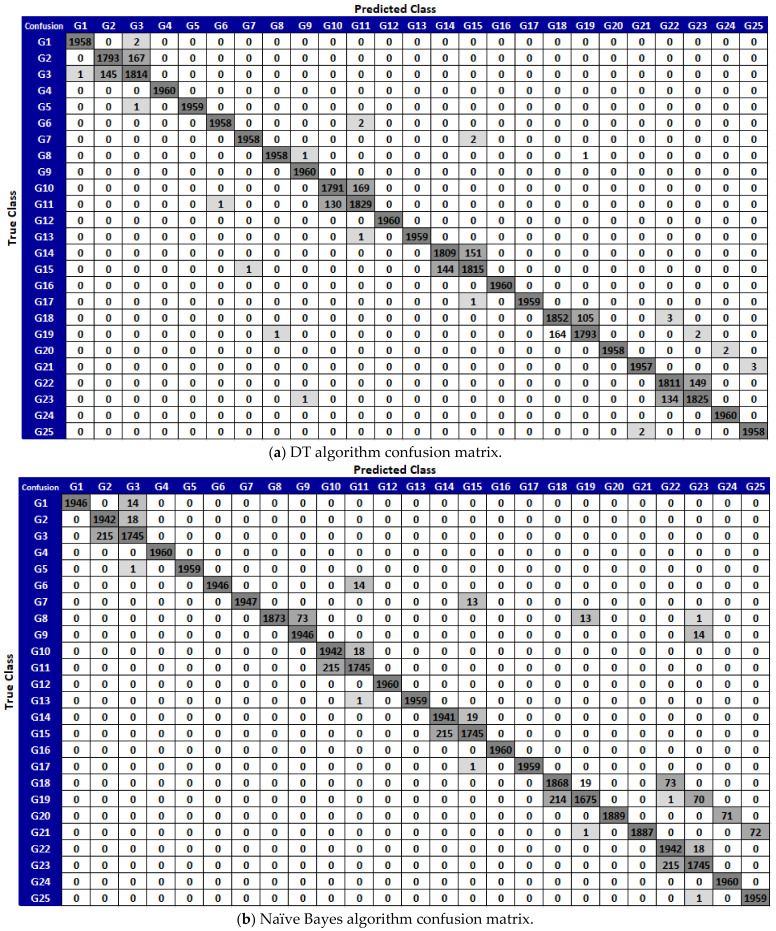

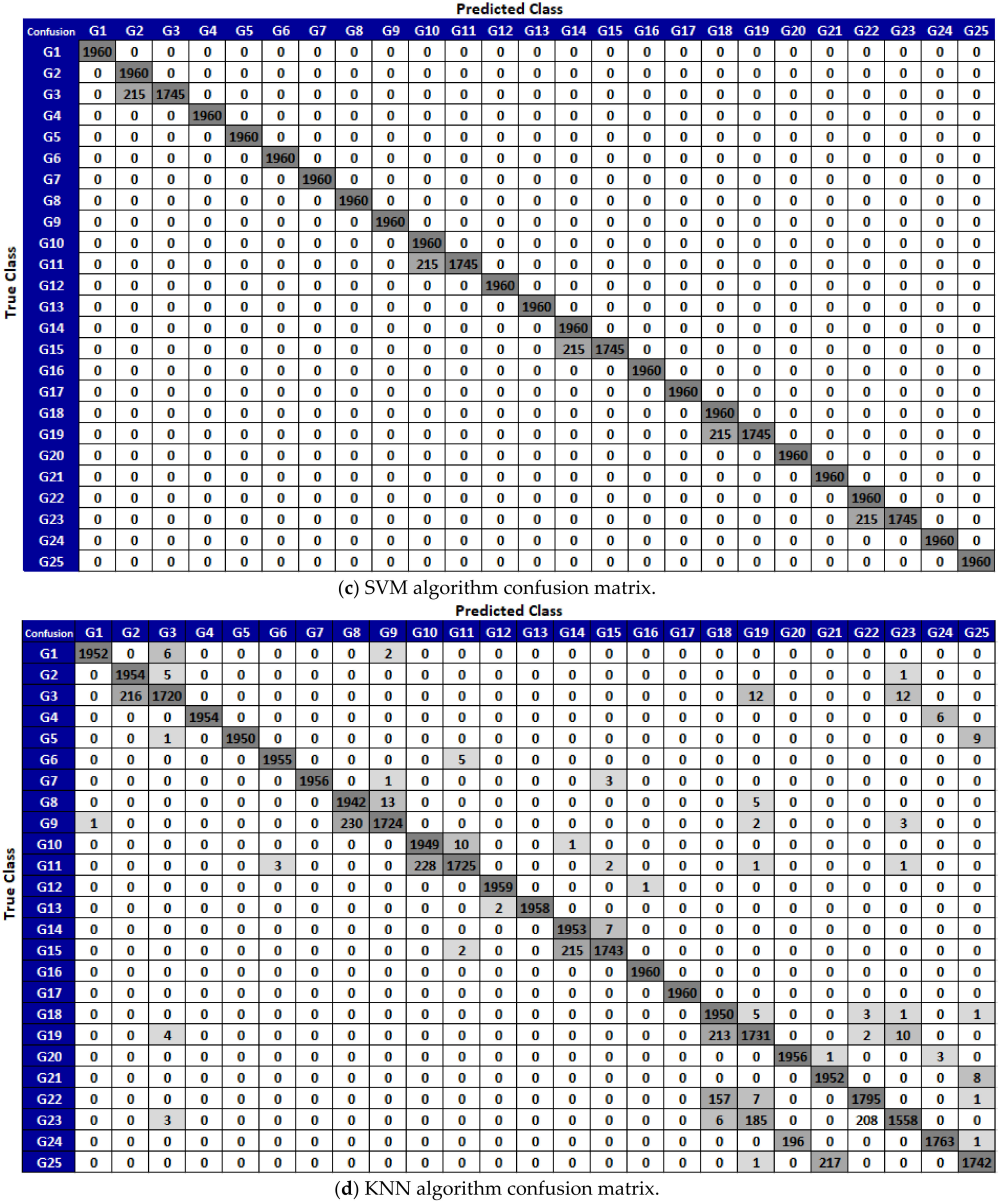
Confusion matrices obtained by operating the (**a**) DT confusion matrix, (**b**) NB confusion matrix, (**c**) SVM confusion matrix, and (**d**) KNN confusion matrix, classification algorithms used to describe the 25 different gestures.

Prior to classification, data were collected preserving 10 s per movement. To ensure the quality and accuracy of the motion signals, 3% of the data in the first and last parts of the received signals were removed since the user had to clearly complete each hand movement. Confusion matrixes were obtained by running 100 iterations of the decision tree (DT), naïve Bayes (NB), support vector machines (SVM), and K-nearest neighbors (KNN) algorithms, whose parameters are given in Table 2. The confusion matrices obtained as a result of the 25 different motion-based DT, NB, SVM, KNN algorithms are given in Figure 7.

A 10-fold cross-validation method was used to increase the performance of the different data sets. First, the data set was randomly and evenly divided into ten parts. Nine out of ten pieces were used to train the classifier, and the remaining data were used in the testing phase. This 10-fold cross-validation was repeated 10 times, resulting in average classification accuracy. The accuracy, precision, recall, geometric mean, F score, and RMSE results obtained for each classification after running 100 iterations are shown in Table 6.

**Figure 8:**
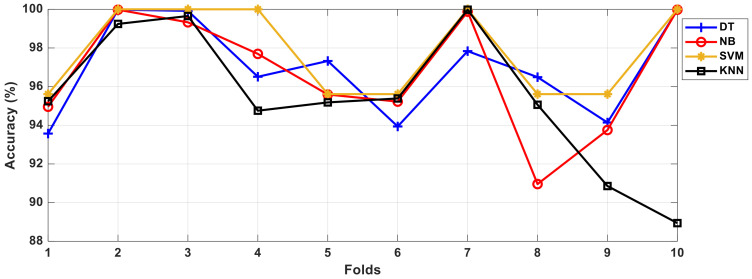
Comparison of the values for Fold-1, Fold-2, Fold-3, Fold-4, Fold-5, Fold-6, Fold-7, Fold-8, Fold-9, and Fold-10 from the 10-fold cross validation method.

The results obtained in the method we performed were obtained with the 10-fold cross-validation method. In Figure 8, for DT, NB, SVM, and KNN classification algorithms Fold-1, Fold-2, Fold-3, Fold-4, Fold-5, Fold-6, Fold-7, Fold-8, Fold-9 The results were calculated by crossing 10 times with Fold-10.

As seen in Figure 8, the two best results for the DT algorithm were calculated with Fold-2, Fold-3, and Fold-10, while the worst results were calculated with Fold-1, Fold-6, and Fold-9. In the NB algorithm, the best results were calculated for Fold-2, Fold-7, and Fold-10, while in the SVM algorithm, the best results were calculated with Fold-2, Fold-3, Fold-4, Fold-7, and Fold-10. For the KNN algorithm, the best results were calculated with Fold-3 and Fold-7, while the worst results were obtained with Fold-10. The times obtained for the feature extraction, training, and testing processes for the DT, NB, SVM, and KNN algorithms are shown in detail in Table 7.

The classification models through which we obtained the accuracy, precision, recall, geometric mean, f-score, and training and test times shown in Table 6 and Table 7 above were implemented in MATLAB version 2020a. The GUI used to connect the smart glove system with the main computer, pre-process the received signals, and collect the data was developed in the Visual Studio C# environment.

## 4. Discussion

**Figure 9:**
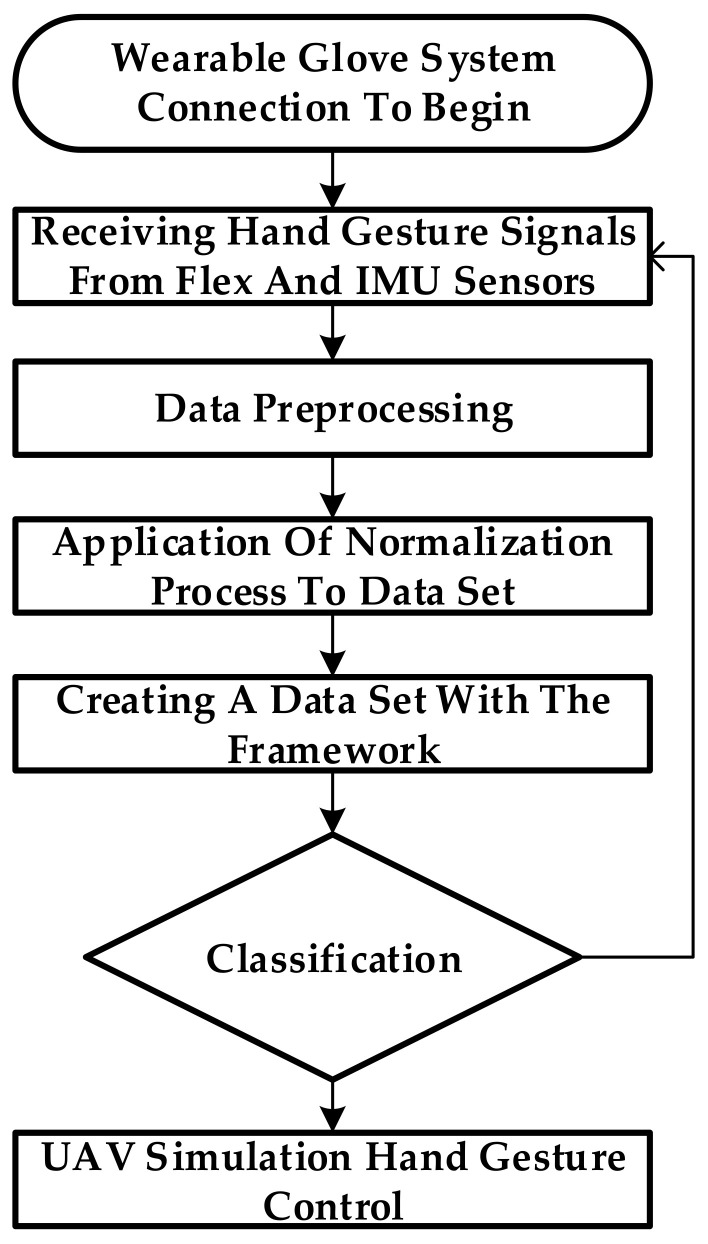
Flowchart showing the workflow process for UAV control with wearable gloves.

In this study, a real-time approach was implemented using a smart pair of gloves to facilitate machine-learning-based human–UAV interactions. The sensing operating combination gloves were designed to be flexible and wearable to provide a safe, comfortable, portable, and affordable solution. A flow chart of the process realized in the background with the user wearing the glove and starting to use the system is shown in Figure 9.

Under the proposed method, with the help of a control mechanism for the UAV flight guidance process, 25 different basic gestures were realized with the two gloves. The 25 movements were created by combining five different gestures from the right and left hands. For the 25 different gestures, 49,000 datasets, each consisting of 11.2 bytes, were obtained from the sensors. After this data set normalization process was applied, the hand gestures were classified using MATLAB’s Classification Learner ToolBox and the decision tree (DT), naïve Bayes (NB), support vector machines (SVM), and K-nearest neighbor (KNN) classification methods; moreover, class was defined. The accuracy rates of the DT algorithm were calculated as 96.96%, the training time was 133.1 ms, and the test time was 2.33 ms. The accuracy rates of the NB algorithm were calculated as 96.91%, with a training time of 183.2 ms and a test time of 26.22 ms. The accuracy rates of the SVM algorithm were calculated as 98.02%, with a training time of 20666.7 ms and a test time of 2.33 ms. The accuracy rates of the KNN algorithm were calculated as 95.77%, with training time of 1096.9 ms and test time of 1.22 ms. The best accuracy rate was achieved with SVM (98.02%), and the best time was achieved with KNN (1.22 ms). Thus, the proposed method successfully recognized 25 different hand gestures with a very good prediction time compared to previous results in the literature. Based on the classification results, the proposed method successfully recognized the hand gestures. Therefore, this system is useable for real-time working performance under the UAV simulation developed in the Unity environment. A comparison between studies in the literature and our study in terms of sensing, processor, number of gestures, number of users, data set size, duration, method, and usage area is clearly provided in Table 8 below.

While 90% hand gesture recognition was achieved for 10 movements in [27], which used similar equipment to that used in our study, the average success rate of our study was about 97%. The authors in [33] used the FCM method for six movements and did not indicate a success rate. In [42], 96.7% accuracy was achieved with 15 ms for six movements, while in our study, an average of 97% accuracy was achieved in 1.22 ms for 25 movements. In [35], a data set with 28800 data for 16 movements achieved 99% success in 67.6 ms, while our study achieved 98% accuracy in 10.8 ms with a data set featuring 49000 data for 25 movements. In [29], for seven movements and 7200 data, 90% accuracy was achieved in 580 ms via the SVM method, whereas in our study, a 98.02% accuracy rate was achieved in 10.8 ms with the SVM algorithm for 25 movements with 49000 data.

## 5. Conclusions

Various solutions have been developed for UAVs, which are increasingly used in many areas today. In this respect, scientific studies on human–UAV interactions are also increasing. While joysticks, remote controls, ground control stations, and mobile devices are generally used to control UAVs, the use of speech, brain–computer interaction techniques, and gesture-based techniques has also increased recently. This paper presents a machine-learning-based, multi-mode, and stable interaction platform that offers mission planning for controlling a UAV with wearable smart gloves developed for human hands. The proposed approach features novel aspects, such as a multi-mode command structure, machine-learning-based recognition, the use of task scheduling algorithms, real-time usage, robust and effective use, and high accuracy rates. Although the performance of the human–UAV interactions via the two wearable smart gloves varies according to the machine-learning-based techniques used and the type of user, it was demonstrated that this system can be used effectively and in real time with an RMSE between 0.14 and 0.21. The effectiveness and efficiency of the proposed approach were demonstrated using a data set that was created by taking 25 different hand movement samples from 20 people for the detection of hand gestures, four different machine-learning-based classification algorithms (decision trees, naive bayes, support vector machines, and k-nearest neighbor), and flight experiments on a real UAV with a total of 10 h of flying. The overall accuracy of the proposed approach was over 98%. These results clearly show that the present system is superior to others in real-time test times and also offers high accuracy and success rates. The test time of the DT algorithm was calculated as 2.33 ms, the test time of the NB algorithm was calculated as 26.22 ms, the test time of the SVM algorithm was calculated as 2.33 ms, and the test time of the KNN algorithm was calculated as 1.22 ms. A comparison between our test results and the results of the studies in the literature is provided in Table 8. This paper contributes to the design of platforms required for the use of wearable technologies in human–UAV interactions.

### 5.1. The Limitations of This Work

The limitations of the proposed method are the resolution of the flex and IMU signals on the wearable smart glove, the time elapsed during communication and algorithm operation under real-time operations, and the size of the data set collected from the users. Since the various movements are similar to each other, it is difficult to obtain motion signals or create a data set without noise.

### 5.2. Future Directions

In the future, we will increase the sizes of the data sets and obtain more motion and command classes, as well as make those classes suitable for working with unmanned systems. Future studies could also develop methods to recognize hand movements or human movements through deep learning. We also intended to control real-time swarm UAV interactions with hand movements in an open field.

## Figures and Tables

**Table 1:** Usage areas, sensing system, and applied study methods in the literature.

Usage Areas	Sensing System	Method	References
Medical Applications	EEG	SVM, CNN	[36,37]
	Force + Flex	SVM, KNN, DT	[40]
	sEMG	LDA	[41]
Fitness and Health	Flex + Pressure Sensor	Visualization	[33]
	EEG	CNN, SVM	[37]
	IMU	DTW	[45]
Knowledge	ERM vibration	ANOVA	[31]
	GSR Sensor − IMU	SVM	[32]
	Flex − IMU	Table of Rules	[29]
	sEMG, IMU	SVM, LDA	[34,35,39]
	EEG	CNN, SVM	[37]
	PGS + Strain	ANN	[4]
UAV	Flex − IMU	FCM	[38]
	Camera	CEA	[8]
	Leap Motion Controller	DL	[30]
	CCD Camera	DL	[26]
	Flex + IMU	STD	[27]
	Spectrum Camera	DL, GCN	[46]

**Table 2:** Parameters of classification algorithms used in the proposed method.

Parameter	DT	NB	SVM	KNN
Type	Fine	Gaussian	Linear	Coarse
Split Criterion	gdi	-	-	-
Maximum Number of Splits	100	-	-	-
Surrogate	off	-	-	-
Distance	-	-	-	Euclidean
Number of Neighbors	-	-	-	100
Distance Weight	-	-	-	Equal
Standardize	-	-	True	True
Kernel	-	Normal	-	-
Support	-	Unbounded	-	-
Kernel Function	-	-	Linear	-
Polynomial Order	-	-	3	-
Kernel Scale	-	-	Auto	-
Box Constraint Level	-	-	1	-

**Table 3:** Hand gestures and UAV task scheduling.

Gestures	UAV Task Scheduling	Gestures	UAV Task Scheduling
1. Steady, Stable	UAV steady, stable	14. Back Up	UAV go back and up
2. Up	UAV fly up	15. Back Down	UAV go back and down
3. Down	UAV fly down	16. Back Right yaw	UAV move back fly towards right
4. Right yaw	UAV fly towards right	17. Back Left yaw	UAV move back at towards left
5. Left yaw	UAV fly towards left	18. Turn right Up	UAV turn right and fly up
6. Forward	UAV go forward	19. Turn right Down	UAV turn right and fly down
7. Back	UAV go back	20. Turn right Right yaw	UAV turn right, fly towards right
8. Turn right	UAV turn right	21. Turn right Left yaw	UAV turn right, fly towards left
9. Turn left	UAV turn left	22. Turn left Up	UAV turn left and fly up
10. Forward Up	UAV fly forward and up	23. Turn left Down	UAV turn left and fly down
11.Forward Down	UAV fly forward and down	24. Turn left Right yaw	UAV turn left, fly towards right
12. Forward Right yaw	UAV go forward fly towards right	25. Turn left Left yaw	UAV turn left and fly towards left
13. Forward Left yaw	UAV go forward fly towards left	1. Steady, Stable	UAV steady, stable

**Table 4:** Flex sensor, IMU sensor, STM32 card specifications.

	Specifications	Value
Adafruit Flex Sensor	Part Length	112.24 mm/4.419 inch
	Active Length	95.25 mm/3.750 inch
	Width	6.35 mm/0.250 inch
	Thickness	0.5 mm/0.2 inch
	Weight	0.5 g
	Flat Resistance	10 K Ohm ± 30%
	Bending Strength	60 k to 110 K Ohm
	Power Rating	0.5 W continuous; 1 W Peak
AltIMU-10 v5	Dimensions	25 × 13 × 3 mm
	Weight	0.8 g
	Input Voltage	2.5 V–5.5 V
	Supply Current:	5 mA
	Gyro	±125, ±500, ±1000, or ±2000°/s
	Accelerometer:	±2, ± 4, ±8 or ±16 g
	Pin Count	48
STM32F103C8T6	Processor	ARM Cortex-M3
	Working Frequency	72 MHz
	Storage Resources	64 K Byte Flash, 20 K Byte SRAM
	Interface Resources	2 × SPI, 3 × USART, 2 × I2 C, 1 × CAN, 37 × I/O ports
	Analog-digital conversion	2 × ADC (12-bit/16-channel)
	RT9193	3.3 V regulator chip, 300 mA maximum output voltage

**Table 5:** Twenty-five different hand movements defined with the help of smart gloves worn on the right and left hands to redirect the UAV.

Gestures	Left Hand	Right Hand	Gestures	Left Hand	Right Hand
1. Steady, Stable 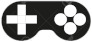	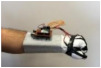	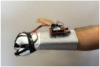	14. Back Up 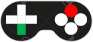	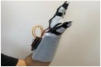	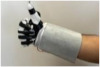
2. Up 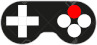	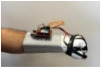	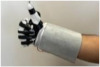	15. Back Down 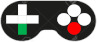	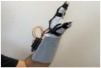	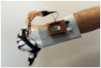
3. Down 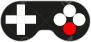	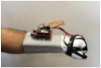	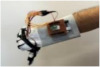	16. Back Right yaw 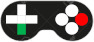	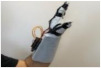	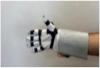
4. Right yaw 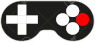	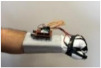	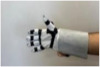	17. Back Left yaw 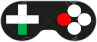	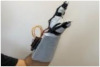	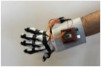
5. Left yaw 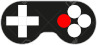	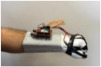	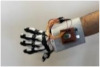	18. Turn right Up 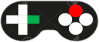	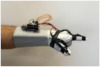	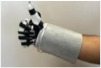
6. Forward 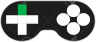	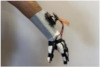	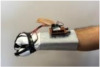	19. Turn right Down 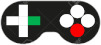	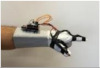	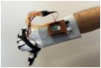
7. Back 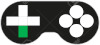	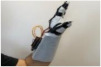	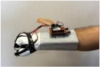	20. Turn right Right yaw 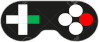	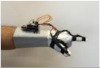	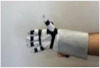
8. Turn right 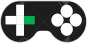	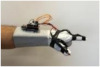	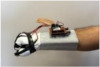	21. Turn right Left yaw 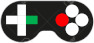	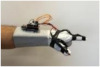	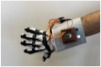
9. Turn left 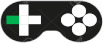	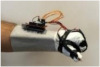	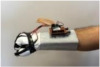	22. Turn left Up 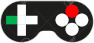	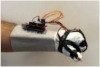	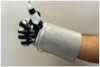
10. Forward Up 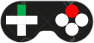	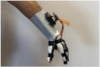	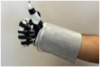	23. Turn left Down 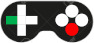	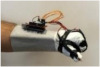	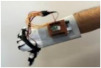
11. Forward Down 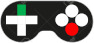	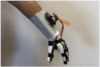	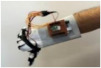	24. Turn left Right yaw 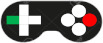	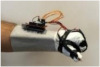	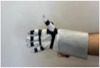
12. Forward right yaw 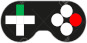	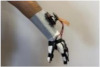	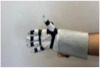	25. Turn left Left yaw 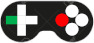	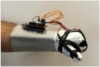	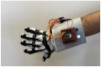
13. Forward Left yaw 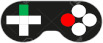	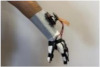	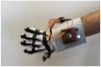	1. Steady, Stable 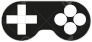	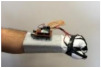	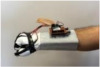

**Table 6:** Accuracy, precision, recall, geometric mean, F-score and RMSE results after 100 iterations.

Classifiers	Statistical Result	Accuracy	Precision	Recall	Geometric Mean	F-Score	RMSE
Decision Tree	Max	96.97	96.96	96.89	96.97	96.96	0.1743
	Min	96.91	96.91	96.84	96.91	96.77	
	Mean	96.96	96.95	96.88	96.95	96.86	
	Std	0.02	0.02	0.02	0.02	0.03	
Naive Bayes	Max	96.92	96.73	96.62	96.82	96.73	0.1757
	Min	96.89	96.70	96.59	96.79	96.67	
	Mean	96.91	96.73	96.62	96.82	96.70	
	Std	0.003	0.004	0.004	0.003	0.01	
SVM	Max	98.02	97.80	97.70	97.91	97.80	0.1407
	Min	97.98	97.78	97.68	97.88	97.69	
	Mean	98.02	97.80	97.70	97.91	97.77	
	Std	0.006	0.003	0.003	0.005	0.02	
KNN	Max	95.77	95.43	95.23	95.60	95.42	0.2056
	Min	95.68	95.32	95.12	95.50	95.30	
	Mean	95.77	95.41	95.22	95.59	95.36	
	Std	0.01	0.02	0.02	0.01	0.02	

**Table 7:** Time of feature extraction, training and test operations for DT, NB, SVM and KNN.

10-Fold CV	DT		NB		SVM		KNN	
	Mean (ms)	Std (ms)	Mean (ms)	Std (ms)	Mean (ms)	Std (ms)	Mean (ms)	Std (ms)
Training Time	133.1	8.2	183.2	0.9	20666.7	98.3	1096.9	42.5
Test Time	2.33	3.82	26.22	78.61	10.83	8.17	1.22	1.24

**Table 8:** Comparison of the proposed system with other studies.

Reference	Sensing	MCU	Gesture	User	Dataset	Training/ Test Time	Method	Accuracy	Usage Areas
[31]	ERM Vibration	Arduino UNO	6	6	360	320/80 (ms)	ANOVA	%86.1	Navigaiton of Swarm of Drones
[32]	GSR + IMU	MCU	-	1	4441	-/20 h	SVM	%94.78	Stress Prediction
[29]	Flex + IMU	Arduino	10	2	-	-	-	%90	Arabic Sign Language Recognition
[33]	Flex + Pressure	Arduino	4	2	10000	-	-	-	Hand Movement
[34]	sEMG	STM32	7	4	7200	-/0.58 sn	SVM	%90	Gesture Recognition
[35]	sEMG	STM32	11	10	7200	-/0.58 sn	HDC	%85	Gesture Recognition
[36]	EEG	PC	8	4	768	8/4.5 (s)	SVM	%70.21–81.05	Mental, Pyscial Practice
[37]	EEG	PZI	8	1	3200	-/30–40 ms	SVM	%96	Human Machine Interface
[38]	Flex + IMU	Arduino Nano	6	1	-	-	FCM	-	Hand Gesture UAV Control
[30]	Leap Motion Controller	Computer	10	7	11062	-	DL	%89.6–96.9	Hand Gesture UAV Control
[39]	EMG + IMU	Computer	8 + 4	10	1600	-/100 ms	LDA	%88.8–92.6	Gesture Recognition
[41]	sEMG	Arduino Mega	9	3	-	320/190 (ms)	LDA	%96.20	Hand Rehabilitation System
[40]	Force + Flex	ARM	16	11	28800	840/67.6 (ms)	SVM, KNN, DT	%99.07, %97.04, %88.52	Hand Rehabilitation System
[26]	CCD Camera	Computer	9	23	6223 pic	-	DL	-	Hand Gesture UAV Control
[4]	PGS + Strain	Computer	3	1	18000	-/100 ms	ANN	%90.4	Hand Gesture
[43]	Spectrum Camera	Computer	6	12	57000	-/15 ms	DL	%96.7	Hand-Action Gesture UAV Control
This Method	Flex + IMU	STM32	25	20	49000	133.1/2.33, 183.2/26.2, 20666.7/10.8, 1096.9/1.22 (ms)	DT, NB, SVM, KNN	%96.96, %96.91, %98.02, %95.77	Hand Gesture UAV Control

## Data Availability

Not applicable.
